# Harvard Odontological Society

**Published:** 1894-11

**Authors:** 

**Affiliations:** Harvard Odontological Society


### Reports of Society Meetings.

### HARVARD ODONTOLOGICAL SOCIETY.

THE regular monthly meeting of the Harvard Odontological Society was held at Young's Hotel, Boston, on Thursday evening, May 31, 1894, at six o'clock, President Eddy in the chair.

*President Eddy.*—We will open our educational work to-night with a paper by our active member, Dr. Arthur H. Stoddard, on "Gutta-percha as a Filling-Material."

(For Dr. Stoddard's paper, see page 709.)

DISCUSSION.

*Dr. Bigelow.*—Dr. Stoddard's method of putting in oxyphosphate fillings with gutta-percha at the cervical wall, I think, is a good one,—that is, the gutta-percha does not disintegrate as fast as the oxyphosphate.

The gutta-percha which Dr. Stoddard prepares I have used for a short time. Its working qualities are good. It seems to be dead, in the sense of not curling. The fact of its having incorporated with it the plaster of Paris makes it desirable as a filling-material, and likely to be quite permanent.

I am a thorough believer in the use of gutta-percha where it can be used with probability of success. I believe we can do more good, save more teeth, and keep our patients in a more comfortable condition by a conservative use of the so-called plastics than we can possibly do by relying entirely on metallic fillings. I remember hearing at the Dental School Dr. Briggs advance very strongly the desirability of using the so-called plastics, particularly gutta-percha. I think he made the statement in the school that at the cervical wall he had greater faith in gutta-percha as a preventer of the recurrence of decay than any other material then in use, and I have become converted to about the same idea.

*Dr. Taylor.*—I have nothing to add, but I have a question to ask. Some time ago I placed some white gutta-percha in the front teeth of a girl fifteen years old; in a short time it had turned very black; the effect was unsightly. It was my first experience with such a result, and I would like to know the cause of this change in color.

*Dr. Reilly.*—I don't know that I can answer Dr. Taylor's question, but will relate my own experience. Some five years ago a paper was read before this Society on this same subject, and at that time I related my troubles with gutta-percha taking on a dark surface in a short time. For warming I was then using soapstone over the gas-flame; I have abandoned it since, and for the past two years have used dentron, heating it (as Mr. Owen suggests) directly over the flame only enough to soften it, and I now find no discoloration. There is one thing I can say for the old way, however, that, while it would discolor, it would wear indefinitely. I use the same instruments and same finishing process.

*Dr. Stanley.*—How did you say you heated it?

*Dr. Reilly.*—I use ordinary gas, Bunsen burner, and hold it above the flame, heating it a very little, depending on the balsam with which the cavity is wiped for adhesion. The pliers take up the heat much faster than the gutta-percha and sink into it when it is warm enough for use. If as much attention be paid to the gutta-percha filling as to gold we shall get better results. I gave up the indiscriminate use of gold fillings ten years ago, and where I put in perhaps three or four a day at that time, now I may do that number in a week.

*Dr. Briggs.*—They are beginning to "out-Herod Herod." I began to teach in 1880 in the Dental School, and was then a disciple of the use of soft fillings, and am still; in fact, I was spoken of as "Briggs, the soft-fillings crank." That, of course, was before the practice became established to the extent that it has now. At that time I was using this same dentron, although it was not known by that name. While very anxious to inculcate the use of gutta-percha, I did not recommend it to the exclusion of all other materials, and I think they have now gone to the other extreme, and are using it too much. It has its place, and is a most useful filling-material, but I do think that there is such a thing as using it too freely, forgetting that there comes a time when other things can be used with better results to the patient.

Perhaps, owing to the fact that I have used it probably longer than any one else in this Society, having begun in 1878, I might give my views on the way to use it. I have always found it better by heating it over the flame, for the reason that one is likely to be more careful in that way than with the soapstone. If you burn it, however, it is utterly ruined, and you must take a new piece. Then, again, I consider it a *sine quâ non* to wipe out every cavity with a solution of resin and chloroform,—that is, perhaps, as important as the gutta-percha itself, and if you do not do that the patients do not get its full value.

I would disagree with the writer in the practice of using a solvent for finishing. I think that any solvent disintegrates it, and the filling may not wear as well. Of course, in finishing, it is harder to do it with a warm instrument than it is with the solvent, which cleans things so nicely; but my experience is that it is better to finish it carefully with a warm instrument. The softening of the surface with the solvent admits of the taking up of coloring matter in the mouth, and it will do it with a poor gutta-percha. It is doubtful if you get such a good gutta-percha as this dentron is, except, perhaps, one which you might make in your own laboratory. I do not like to make invidious distinctions between manufacturers, but the probability is that no other one would ever make it as carefully as that is made. Where it is not so thoroughly prepared it is more liable to disintegrate and undergo fungus growth on the surface; hence the softening and discoloration, and consequent annoyance to yourself and patient.

I am a great believer in its use, and if I did not use it for anything else, I feel that I could not get along very well without it in the filling of root-canals. I know that there has been of late an advocacy of the use of the cements for root-canals, but the idea is with me that if a healthy root-canal is filled thoroughly almost anything will do; if the root-canal, however, is diseased, and you have to be sure that you fill through the apex of the root, you can place gutta-percha up through it without causing any inflammation, and you cannot do that with any other filling-material and be sure that it will not set up an irritation. I have many times cured old fistulæ by filling them with gutta-percha, and I have been so successful, even where the history of the case, extending sometimes over ten or twelve years, has been of chronic abscess, that I am a firm believer in the compatibility of gutta-percha with the soft tissues, which is the great issue in the filling of root-canals.

*Dr. Clifford.*—May I ask Dr. Briggs why he prefers heating it over the flame to the soapstone?

*Dr. Briggs.*—Because, if you place it on the soapstone, it is left there while you are operating, and, if not all used, I doubt if you throw it aside. Over-cooking is just as bad as burning it. The idea is to heat a piece of the right size, and to heat it just enough. I put it near the flame at first and gradually raise it as it warms. As Dr. Reilly says, the pliers sink into it, and you can easily tell when it is warm enough to use. If I see that it gets over-cooked, as it often will, I throw that piece away and take another. In placing it on the soapstone one would be inclined to run into the danger of allowing it to remain there too long.

*Dr. Clifford.*—What kind do you use?

*Dr. Briggs.*—I have used the same kind of gutta-percha since 1878. I was then with Dr. Williams, and this Mr. Owen came into our laboratory about ten or twelve years after that and was taught how to make it, and, I think, perhaps, at my suggestion, he began to make it to sell to others. I am willing to pay him for it, because I know that I could not do it, or have it done,—as I assume he continues to make it,—for any less price. In order to get the best results in the manufacture of gutta-percha one should work with only a small amount of heat and material; therefore, but a small quantity can be made at a time.

*Dr. Grant.*—I want to ask Dr. Stoddard if he ever found, after keeping his gutta-percha some time, that it grew gradually harder,—that is, where it is made with a solvent and then dried?

*Dr. Stoddard.*—I have noticed it in the mouth. After gutta-percha fillings have been inserted for two or three months and you attempt to take one out, you will find it is quite hard.

*Dr. Grant.*—I asked because I used to try to make gutta-percha, and did make it until I could buy an article which suited me. I was taught how to make it by Dr. Keep, and his great point was to condense it,—that is, to roll or knead it until it cannot be rolled any longer. Where it is made with a solvent I found that it is likely to be a little porous, although, as Dr. Stoddard said, it is very hard. I made some, ten or twelve years ago, that was almost as hard as glass, and I thought then, if I could heat it and place it in the cavity and get it to resume that degree of hardness, I should have almost a perfect filling. You could not get it as hard, it seemed to me, if you put it through a rolling-mill. Looking at this I thought I recognized some features about it that I had seen before.

*Dr. Stoddard.*—I use it principally as a temporary stopping. I made it myself, not from any formula, but from experience. I tried it first by using chloroform, and found that that did not dissolve it thoroughly, and it evaporated too quickly, so that I could not stir in the powder. I then tried the oil of cajuput, which seemed to dissolve it very nicely in connection with the chloroform.

*Dr. Reilly.*—I would like to ask Dr. Stoddard if Mr. Owen suggested the use of any solvent for finishing the surface of his dentron?

*Dr. Stoddard.*—I knew when I made the statement it was likely to meet with opposition. Of course, I have not had so much experience as Dr. Briggs, but I have watched the fillings very carefully with that in view. For several years I did not use a solvent, and I have watched them carefully to see if I could detect any difference between fillings put in under the different conditions, and I cannot see that there is a particle of difference in the wear. I know the theory is that a portion of the surface does disintegrate under the solvent, but practice does not prove it to me as yet.

*Dr. Reilly.*—Do you burnish after that? I ask because I have talked with Mr. Owen to find out if there was not some way to get the filling to show the original surface that he gets on his guttapercha. It is quite a fair polish. There might be saving of time if, after using a solvent to cut down the surplus, one could use the burnisher and get a finished, glossy surface. Finishing with the instrument takes a great deal of time. Of course, it is very readily done with the solvent. Perhaps Dr. Stoddard has tried that way?

*Dr. Stoddard.*—I have not used it in just that way. I always use a very little of the solvent, not so much for finishing off the filling as for smoothing the overhanging edges and fragments; I then finish off with an instrument and wipe with a dry tape after I get through.

*Dr. Reilly.*—Your last treatment, then, is a dry tape?

*Dr. Stoddard.*—Yes.

*Dr. Hitchcock.*—On looking over my records in regard to gutta-percha fillings, some four years ago, I found that out of over thirteen hundred there were only two cases that failed,—a result that one could not get with any other filling-material. Since then I have put in perhaps two thousand more. I do not use resin, but dry out the cavity as perfectly as possible. I frequently use a solvent in finishing,—simply a very small pledget of fibrous paper dipped in chloroform, and finally completing with a burnisher. I have had but very few cases of this discoloration, none of them, I think, in the front part of the mouth.

*Dr. Clifford.*—What kind of gutta-percha do you use?

*Dr. Hitchcock.*—In hidden situations I use the pink. This sometimes lasts fifteen to twenty-five years. I have known it to last on the grinding surface for ten years. When it is worn, it is a matter of two or three minutes to remove the outer portion with a bur and place on more. Decay under it is less than under metallic fillings.

*Dr. Werner.*—I am decidedly on the other side; and, after what has been said, I must speak from my point of view. Gutta-percha with me has a very limited place as a filling-material, and when I hear of dentists putting in two thousand and thirteen hundred fillings in a few years, added to what the essayist has said, I should be inclined to laugh were it not for my sympathy with the patients. I would like to know what kind of surfaces they were. Were they approximal and approximo-coronal?

*Dr. Hitchcock.*—All kinds.

*Dr. Werner.*—I would like to see an approximo-coronal surface filled with gutta-percha that would be good for longer than a few months; good for comfort, good for cleanliness, or good for grinding. I am a thorough believer in full contour fillings in all approximo-coronal surfaces, and gutta-percha is not a good substance to fill such cavities with. It is not clean; it will make teeth sensitive, and when pressure comes on it, it cannot remain perfect. I do not want to say too much for fear I should go to the other extreme.

*Dr. Briggs.*—Keep right on,—you are there already.

*Dr. Werner.*—But in my opinion plastic materials, especially gutta-percha, are on the decrease in proportion as we have skilful gold and amalgam operators.

*Dr. Reilly.*—I would like to ask Dr. Werner to define an approximal cavity.

*Dr. Werner.*—An approximo-coronal cavity, such as I refer to, extends from the cervical wall to and including part of the grinding surface. I think to fill such a cavity with gutta-percha is folly.

*Dr. Reilly.*—At any age?

*Dr. Werner.*—As a treatment, I admit it has its advantages.

*Dr. Briggs.*—What is any operation but a treatment?

*Dr. Werner.*—But we have better things that we can use for treatment. By using the cements we obtain a hardening effect, and as long as the cement lasts you have a clean surface and a filling-material that is non-discoloring, non-fungi producing; and when the cement is worn, scrape it away and add more, and you are making your dentine and tooth harder and preparing it for a permanent filling.

*Dr. Reilly.*—Will Dr. Werner please tell us how many fillings he has ever made of dentron in approximal cavities?

*Dr. Werner.*—I think dentron is the best gutta-percha preparation we have. I have used two bottles of it in perhaps six years.

*Dr. Reilly.*—That is not answering my question,—How many approximal cavities have you filled with dentron?

*Dr. Werner.*—I should never fill an approximo-coronal cavity permanently with any kind of gutta-percha.

*Dr. Reilly.*—Have you ever filled an approximal cavity with gutta-percha as a temporary filling?

*Dr. Werner.*—As a treatment, yes. I filled one this afternoon at half-past four—a small cavity in the left superior lateral incisor—with dentron. Neither the patient nor myself had time for a gold filling. That will arrest decay and may possibly last a couple of years; but if I could have made a non-cohesive gold filling, and taken perhaps a little more than twice the length of time it took with gutta-percha, it would have lasted much longer.

*Dr. Hitchcock.*—I hope Dr. Werner does not think we would take an approximal cavity involving half the crown and fill that entirely with gutta-percha? In cases which extend over the crown, we expect them to suffer from attrition; but five minutes used in replenishing them makes a filling that will give another three or four years' wear. I have had to take out a large number of gold, amalgam, and cement fillings on account of the continuation of decay. The greater part of those fillings have been replaced with gutta-percha with few instances of recurrences of decay. I had a case this week where, of thirty-seven metallic fillings, nearly all were removed on account of decay and replaced with gutta-percha. From former experience with such cases I expect to find the caries stopped.

*Dr. Briggs.*—I don't want Dr. Werner to go on record as having spoken in a slighting manner of the temporary virtues of soft fillings without asking him how long a time he has put his much-vaunted contour fillings to test,—whether he has practised full contour filling long enough to say that they are more than temporary fillings?

*Dr. Werner.*—Strictly speaking, I suppose all operations on the teeth are temporary; certainly all gutta-percha fillings are. The essentials of what we call a permanent filling consist in a material that will not discolor and that will retain its full contour for a number of months or years. How long will your gutta-percha filling last on the labial, proximal, or grinding surface with a patient who is cleanly, uses the brush, and grinds with the tooth?

*Dr. Hopkins.*—I have one in my mouth that has been there for fifteen years.

*Dr. Werner.*—I have a right to go on record as I choose. I would like to see gold and gutta-percha tested in two cavities of approximo-coronal surfaces. I will fill one with gold, and you who believe so thoroughly in gutta-percha the other,—they shall have the same environment,—and we shall keep a record. We will then see which of the two proves most serviceable to the patient, considering the length of service, comfort, and money paid.

*Dr. Briggs.*—Dr. Werner has not answered my question as to how long his contour fillings have lasted?

*Dr. Werner.*—Ever since I graduated from the Dental School I have been a thorough believer in restoring full contour. I have seen contour operations that have lasted thirty years.

*Dr. Briggs.*—They were not yours, though.

*Dr. Werner.*—I think the gentleman next to me (Dr. Page) has a contour gold filling in one of his teeth which I put in eighteen years ago. I don't mean to say that gutta-percha should not be used at all; it has its place, and that place with me is very limited. And when you speak of making thirty-seven gutta-percha fillings in one mouth, such seems to me ridiculous. I cannot conceive of such a case in my practice. When an approximo-coronal surface is thoroughly and skilfully restored with gold, you will have comfort and security for ten, fifteen, or twenty years; and when it decays at its weakest place (the cervical wall), then patch it with gutta-percha or alloy. With me it is largely a question of skill in regard to the success of gold fillings, which you say do not prevent decay.

*Dr. Stanley.*—Two things are to be considered in connection with this matter,—first, there is no one material that is the best to use in all places. In many places gutta-percha is infinitely superior to gold; second, even though the best material for a given cavity is used, and used unskilfully, it does not save the tooth. When a soft filling is required, I find I get the best results from a combination of gutta-percha and cement; in many cases gold is not to be compared with it in the saving of teeth. The cement will harden the tooth so that at some future time a gold filling may be placed in.

*Dr. Reilly.*—I think Dr. Werner's statement, that he would like to see any one put in an approximal filling that will last over two or three months, ought not to be allowed to pass. I will place in a filling with Fowler's temporary stopping that will last longer than that.

*Dr. Werner.*—What kind of a contour do you get with such fillings?

*Dr. Reilly.*—I am not looking for contours, I am looking for the salvation of the teeth. I don't care to wedge teeth half an inch apart and build them up with gold to make them look pretty, and then find out my mistake after death of the pulp. Much harm may result from the injudicious use of gold. I am not an extremist on soft fillings; I make use of all the materials, but, as Dr. Stanley says, my best experience has been with combination fillings.

*Dr. Stanley.*—I had a family of children whose teeth were very poor, and I think bad judgment had been exercised in the handling of them. Metallic fillings had been put in,—some gold and some amalgam; these I removed and filled the best I could with gutta-percha, in combination with cement, and sometimes entirely with gutta-percha. There were some small cavities on the crowns of bicuspids, and I used dentron in those places. That was over a year ago. I have recently seen them, and they show no signs of wear at all. I would just as soon think of filling those teeth with Portland cement as with gold.

Subject passed.

*President Eddy.*—Dr. Werner will show models of a case before and after full contour restoration on molars and bicuspids.

*Dr. Werner.*—I don't know, Mr. President, after the expression of opinion of some of the gentlemen here to-night that they will be very much interested in seeing this case. They are the models of a case which I expected to have operated upon at the clinic, but I could not get the necessary spreading apart of the two bicuspids in time. It has been done since, and any one who is interested may see the result. They speak for themselves. Of course, if I had filled those places with gutta-percha, they would not have answered the object that the patient had in coming to the dentist. The patient came to the dentist to have the teeth filled, and to get as much comfort from those teeth as possible. Now these surfaces are restored to full contour, and they are complete. We will keep a record of them and see how long they last.

Adjourned.

HENRY L. UPHAM, D.M.D.,
*Editor Harvard Odontological Society.*

